# Ayurvedic Herbal Preparation Supplementation Does Not Improve Metabolic Health in Impaired Glucose Tolerance Subjects; Observations from a Randomised Placebo Controlled Trial

**DOI:** 10.3390/nu13010260

**Published:** 2021-01-18

**Authors:** Diederik Esser, Juri Matualatupauw, Ric C. H. de Vos, Ron Wehrens, Jos van der Stappen, Ingrid van der Meer, Lydia A. Afman

**Affiliations:** 1 Division of Human Nutrition & Health, Wageningen University, 6708 WE Wageningen, The Netherlands; diederik.esser@wur.nl (D.E.); jurimatualatupauw@gmail.com (J.M.); 2 Business Unit Bioscience, Wageningen Research, 6708 PB Wageningen, The Netherlands; ric.devos@wur.nl (R.C.H.d.V.); ron.wehrens@wur.nl (R.W.); ingrid.vandermeer@wur.nl (I.v.d.M.); 3 Department of Clinical Chemistry, Canisius Wilhemina Hospital, 6532 SZ Nijmegen, The Netherlands; j.v.d.stappen@cwz.nl

**Keywords:** type 2-diabetes, Ayurvedic herbal, adipose tissue, whole genome gene expression

## Abstract

The increased usage of alternative Ayurvedic treatments as potential health-beneficial therapies emphasizes the importance of studying its efficacy in sound placebo-controlled intervention trials. An example of such a traditional Ayurvedic herbal preparation is Mohana Choorna, a mixture composed of 20 different herbs and used to prevent and treat type 2-diabetes (T2D). We studied the efficacy of “Mohana Choorna” on T2D-related parameters in subjects with impaired glucose tolerance. In a double blind, placebo-controlled cross-over trial, 19 overweight (BMI > 27 kg/m^2^) subjects aged 50–70 years with an impaired glucose tolerance received two four-week interventions, i.e., herbal or placebo with a four-week wash-out between interventions. HbA1c, glucose, insulin, triglycerides, cholesterol, blood pressure and augmentation index were measured before and after both interventions at fasting and during a glucose tolerance test. After both interventions, urine was collected to measure treatment exposure using LCMS-based metabolomics and whole genome gene-expression in adipose tissue of 13 subjects. The herbal intervention did not affect plasma glucose triglycerides, cholesterol, blood pressure or the augmentation index but showed a trend towards an increased insulin, HOMA-IR and postprandial insulin levels (*p* = 0.054, *p* = 0.056 and *p* = 0.095 respectively). An increase in expression of inflammation-related gene sets in adipose tissue was observed after the herbal intervention compared to placebo. Urine metabolomic analysis did not reveal a correlation of the presence of specific plant metabolites with “health markers”. Our findings suggest that there is no substantiating evidence to claim that four weeks’ use of the Ayurvedic herbal supplement Mohana Choorna beneficially affects glucose homeostasis.

## 1. Introduction

Type 2 diabetes (T2D) is a chronic metabolic disorder and rates have increased rapidly in the last couple of decades [1]. T2D has a genetic component, but lifestyle factors such as diet and exercise play an important role in the onset and one of the major determinants of T2D is obesity [1]. Metabolic disorders such as T2D are very complex and are often the result of a misbalance in the interplay between several organs such as liver, adipose tissue and muscle. Numerous drugs are currently prescribed to treat T2D, each targeting different organs and/or signaling routes. As an example, metformin suppresses hepatic gluconeogenesis via AMPK activation, thiazolidinediones (TZDs) activate PPARs and DPP4-inhibitors inhibit glucagon release via incretin release. Other type of drugs targeting metabolic health-associated conditions such as dyslipidemia or hypertension are also often prescribed.

Many plant compounds are pharmacologically active and have been the basis for medical treatments. The correlation between the ethno-medicinal usage of medicinal plants and modern medicines discovered from those plants has been studied by Fabricant and Farnsworth [2]. Based on their analysis, 88 single chemical entities isolated from 72 medicinal plants have been introduced into modern therapy, many of which have the same or a similar therapeutic purpose as their original ethno-medicinal use. Some of these plant-derived compounds are still widely used as single agents or in combination formulations in prescription drugs.

Traditional medicine by herbal treatments is still widely practiced today and is used in many cultures for the maintenance of health and for the prevention, improvement or treatment of many diseases including T2D. From animal studies there are indications that some particular type of herbs may lower glucose and lipid levels in the blood. For example, consumption of Monordica charantia (bitter melon) extracts increased insulin sensitivity and lowered blood glucose in mice [3]. Incorporation of coriander into the diet lowered blood glucose levels in diabetic mice [4]. Consumption of Fagopyrum esculentum (Buckweat) Morus alba (white mulberry) leaf extract is able to lower plasma cholesterol levels in rats [5,6]. Nasturtium officinale (Watercress) may have beneficial effects on vascular function [7]. However, almost all scientific evidence is based on results of in vitro or animal models. Some human parallel-designed intervention studies with herbs have been performed but a meta-analyses reported that due to methodological deficiencies and small sample sizes the authors were unable to draw any conclusions regarding the efficacy of the treatments [8]. Another important aspect of herbal supplement use is the combination of different herbs. Where most research in vitro and in animal models has been performed with individual herbal compounds, herbal supplements available in real life are often composed of a combination of herbs. Similar to diets, a combination of herbs can be expected to target different cellular pathways and signaling routes. A powerful strategy to understand how bioactive compounds may affect cellular processes is by using transcriptomics. The general access to genome-wide screening techniques, besides the essential physiological measurements, allows the identification of affected metabolic pathways. Likewise, metabolomic analysis of plasma [9] or urine [10] samples can be used to check for the actual uptake of and exposure to herbal metabolites and to correlate urine metabolite profiles to T2D parameters in order to identify possible bioactives.

Many commonly used commercially available herbal mixtures derive from Ayurveda and have their historical roots in the Indian subcontinent. Although not used in regular medical practice, the use of these Ayurvedic herbal treatments is also increasing in Western countries [11]. An example of such a traditional Ayurvedic herbal preparation is Mohana Choorna (MC), which is currently used for the treatment of early stages of T2D. The question remains whether these herbal treatments are really able to prevent T2D or improve clinically accepted T2D-related parameters. The use of such herbal preparations in disease treatment is therefore viewed with great skepticism by the scientific community. However, the increased usage of these alternative Ayurvedic treatments emphasizes the importance of studying the efficacy of these herbal supplements following an internationally accepted scientific approach with proper controls.

We therefore aimed to study the efficacy of the Ayurvedic herbal product “Mohana Choorna” on T2D-related biochemical parameters in subjects with impaired glucose tolerance by conducting a randomized crossover placebo-controlled trial. Beside these physiological measures, we also aimed to identify potentially affected metabolic pathways in adipose tissue samples to understand how these herbs may potentially affect metabolic health.

## 2. Materials and Methods

### 2.1. Ethics Statement

All subjects gave written informed consent and the study was approved by the Medical Ethics Committee of Wageningen. The study was conducted according to the principles of the Declaration of Helsinki, in accordance with the Medical Research Involving Human Subjects Act (WMO) and registered at Clinical Trials.gov (identifier NCT NCT02065271).

### 2.2. Subjects

Overweight (BMI > 27 kg/m^2^) male and postmenopausal female subjects between 50 and 70 years old with an impaired glucose tolerance were recruited. Impaired glucose tolerance was determined by either a 75 g two-hour oral glucose tolerance test (OGTT) with glucose levels >7.8 mmol/L and/or a fasting glucose > 6.1 mmol/L. All subjects were non-smoking, had a stable body weight and were not diagnosed with a chronic medical condition or high blood pressure (systolic > 160 mmHg and/or diastolic > 100 mmHg). Subjects were not allowed to use any medication or food supplements known to interfere with glucose homeostasis. Subjects were not allowed to participate if they used medication, known to interact with Hypericum perforatum (St. John’s Wort). Female participants were not allowed to use any hormone treatment and the menstrual cycle had to be absent for more than one year.

### 2.3. Study Design

**Figure 1:**
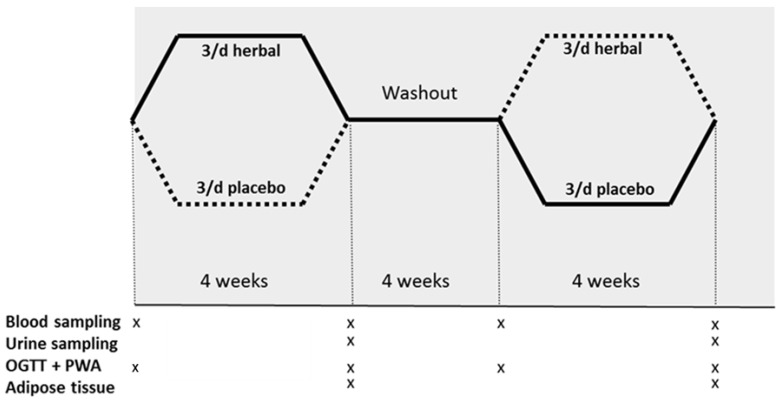
Study design: double-blind, placebo-controlled cross-over 4-week intervention trial in 22 middle-aged subjects with impaired glucose tolerance. Subjects consumed, in random order, four weeks herbal and four weeks placebo supplements three times a day. OGTT; oral glucose tolerance test, PWA; pulse wave analysis.

The study was a randomized, double blind, placebo-controlled cross-over intervention trial in which subjects received two interventions, e.g., an intervention with a herbal mixture and an intervention with a placebo supplement (Figure 1). During the 4 week intervention, subjects consumed three times a day one capsule with either the herbal supplement or the placebo before a meal. Subjects received both interventions in a random order with a wash-out period of 4 weeks between both interventions. Fasting blood samples were collected and vascular measurements were performed before and after each intervention period. Subjects were also subjected to an 75 g oral glucose tolerance test (OGTT) before and after each intervention period (Glucomedics, Beldico, Duiven, the Netherlands). Blood samples were collected 30, 60, 90 and 120 min after consumption of the glucose drink to measure glucose and insulin responses during the OGTT. We also measured vascular function 60 and 120 min after the OGTT. After both intervention periods, we collected an adipose tissue biopsy and a 24 h urine sample. Adipose tissue collection was voluntary and participants were able to participate in this study without donating a biopsy.

Supplements were distributed every two weeks. To assess compliance extra supplements were distributed and subjects were asked to bring back left-over supplements and to keep a diary to report deviations from the study protocol. Subjects were asked to keep the same dietary and physical activity habits as before the study to remain a stable weight. The weight of the participants was measured every two weeks. On the day prior to each testing day, subjects had to refrain from alcohol or exercise and were not allowed to eat or drink anything except water after 08.00 pm.

### 2.4. Herbal and Placebo Supplement

The herbal mixture (Mohana, Choorna, European Ayurveda Centre, Witharen, The Netherlands) comprised a mixture made from twenty herbal species (Table 1). This particular mixture was selected because it was developed as a treatment for T2D and because of the potential efficacy of the individual herbal compounds described in the literature (Table 1). Placebo capsules were filled with microcrystalline cellulose (Fagron, Capelle aan den Ijssel, The Netherlands). Both herbal and placebo capsules contained 500 mg and were identical in size and color (Fagron, Capelle aan den IJssel, The Netherlands). Herbal supplements were screened for any potential harmful toxins (Europroxima BV, Arnhem, The Netherlands). This rapport was included in the medical ethical dossier and all tested mycotoxins and microorganisms were within acceptable levels.

### 2.5. Metabolic Parameters and Differentiated Leukocyte Count

Liver function parameters alanine aminotransferase (ALAT) and aspartate aminotransferase (ASAT) were monitored weekly in order to detect potential adverse changes during the first supplementation period. Fasting blood samples were collected before and after each intervention period. For HbA1c, whole blood was directly stored at 4 °C after collection and analyzed within 5 days by the clinical chemistry laboratory (Canisius-Wilhelmina hospital, Nijmegen, The Netherlands). Plasma glucose, insulin, triglycerides (TG), free fatty acids (FFA), total cholesterol, ALAT and ASAT were also analyzed by this laboratory. A differentiated leukocyte count was performed by a “point of care” analyzer (WBC DIFF analyser, Hemocue, Eindhoven, The Netherlands).

### 2.6. Measures of Vascular Function

Vascular measurements were performed under fasting conditions and 60 and 120 min after the OGTT. All vascular measures were performed in a supine position after 5 min of rest in a temperature-controlled room. Brachial systolic blood pressure (SBP) and diastolic blood pressure (DBP) were assessed automatically (DINAMAP^®^PRO 100) for 6 min with a 2 min interval. Heart rate-corrected augmentation index (Aix), a measure of wave reflection and arterial stiffness, was assessed by a pulse wave analysis (PWA) of the radial artery (SphygmoCor^®^CP system, ATcor Medical, Sydney, NSW, Australia) as described previously [36].

### 2.7. Adipose Tissue Collection and RNA Isolation

Subcutaneous adipose tissue samples were collected voluntarily at the end of each intervention period. Of the 13 subjects, adipose tissue biopsies were collected after an overnight fast by needle biopsy from the periumbilical area under local anesthesia. The samples were rinsed with PBS to eliminate blood. Cleaned samples were immediately frozen in liquid nitrogen and stored at −80 °C. Total RNA was isolated from adipose tissue using Trizol reagents (Invitrogen, Breda, The Netherlands) and RNeasy Mini Spin columns (RNeasy mini kit, Qiagen, Venlo, The Netherlands). RNA was quantified (Nanodrop ND 1000, Nanodrop technologies, Wilmington, DE, USA) and integrity was checked by an Agilent 2100 Bioanalyser with RNA 6000 nanochips (Agilent Technologies, South Queensferry, UK). Samples passed the RNA quality control if RIN > 7.

### 2.8. Microarray Processing and Analysis

Microarray analysis of adipose tissue was performed for all 13 individuals after 4 weeks supplementation of placebo or herbal treatment, resulting in a total of 26 microarrays. Total RNA was labelled using a one-cycle cDNA labelling kit (MessageAmp™ II-Biotin Enhanced Kit; Ambion Inc., Nieuwekerk a/d IJssel, The Netherlands) and hybridized to GeneChip^®^ Human Gene 1.1 ST Array targeting 19,738 unique gene identifiers (Affymetrix Inc., Santa Clara, CA, USA). Sample labelling, hybridization to chips and image scanning were performed according to the manufacturers’ instructions. MADMAX pipeline was used for statistical analysis of microarray data [37]. Microarrays were analyzed using the reorganized oligonucleotide probes, which combine all individual probes for a gene [38]. Expression values were calculated using the robust multichip average (RMA) method and normalized by quantile normalization [39,40]. Genes with normalized signals >20 on >10 arrays were defined as expressed and selected for further analysis. Microarray data are registered as GSE164934 in the Gene Expression Omnibus. Data were further analyzed with gene set enrichment analysis (GSEA) [41] to identify regulated gene sets. Gene sets with an FDR Q-value < 0.25 were visualized in Cytoscape (Cytoscape 2.8.3) and gene sets containing overlapping genes were cluster-based on an overlap coefficient cut-off of 0.5.

### 2.9. Urinary Metabolomics

An essentially untargeted LCMS-based metabolomic analysis was performed on 24 h urine samples collected after each intervention. Samples were extracted as described before [10] and subsequently analyzed using high resolution LC-MS. An UltiMate 3000 U-HPLC system (Dionex) was used to create a 45 min linear gradient of 5–75% acetonitrile in 0.1% FA in water at a flow rate of 0.19 mL min-1. Of each extract, 5 µL was injected and compounds separated on a Luna C18 column (2.0 × 150 mm, 3µm; Phenomenex) at 40 °C. A Q Exactiveplus-Orbitrap FTMS (Thermo) was used to detect eluting compounds: the FTMS was set to detect masses at a resolution of 70,000 in negative electrospray ionization (ESI) mode over a range of *m/z* 90–1350. Raw LCMS data files were subsequently processed in an untargeted manner using the dedicated Metalign-MSClust workflow described previously [42], including unbiased peak picking, alignment and assembling of mass signals likely derived from the same metabolite. This unbiased processing resulted in 3498 putative compounds detected in ESI-negative mode, each characterized by a specific in-source mass spectrum, including the putative molecular ion, its isotope(s) and possible adducts and in-source fragments, at a specific retention time. The data of this untargeted metabolite profiling was subsequently used for statistical analyses to identify possible urine metabolite markers for (I) actual herbal intake and (II) T2D parameters.

### 2.10. Statistical Analysis

Statistical analysis was performed using linear mixed models for repeated measures (PASW statistics 18.0.3, IBM). The effect of the intervention was determined using treatment (herbal or placebo), time (before or after intervention) and the interaction treatment*time as a fixed effect. Baseline values were included in the model as a covariate. The difference in OGTT responses of plasma glucose and insulin due to the intervention was analyzed by comparing the concentrations at 120 min after the OGTT and by calculating the incremental area under the curve using the trapezoidal method [43]. Effect of the intervention on the OGTT response curves was also determined by comparing the change (∆week4-week0) in the response curve between interventions by using the “interaction treatment*postprandial time” as a fixed effect. A least significant difference (LSD) posthoc analysis was performed if significant differences were detected by the model. A *p* value < 0.05 was considered to be significant.

Differences in adipose tissue gene expression from the microarray analyses between herbal and placebo treatment were calculated from the individual signal-to-log ratios (SLR). A change (herbal vs. placebo) was considered significant if the *p* value in a paired t-test with Bayesian correction was <0.05 (Limma) [44]. Gene sets with a false discovery rate (FDR) Q-value < 0.25 were defined as significantly regulated.

The metabolomics data from the urine samples were analyzed using the Wilcoxon signed-rank test, the non-parametric equivalent of a paired *t*-test. This is because of the large number of non-detects in the data, due to metabolites being below the detection limit. These non-detects have been replaced by the lowest value in the data matrix, a procedure that for parametric tests would lead to problems but that does not change the results for non-parametric tests. Afterwards, *p* values were corrected using the Holm correction [45].

To assess a potential association between changes in the herbal-metabolites present in the urine and metabolic parameters related to glucose handling such as plasma HbA1c, plasma glucose, OGTT AUCglucose, insulin, OGTT AUCins and HOMA-index, a multivariate analysis was performed. The multivariate analysis method used was PLS regression [46,47]. This method compresses the information in the independent variables (e.g., the urine metabolites) in a subspace, relevant to the prediction, and then performs least squares regression.

## 3. Results

### 3.1. Subject Characteristics

**Figure 2:**
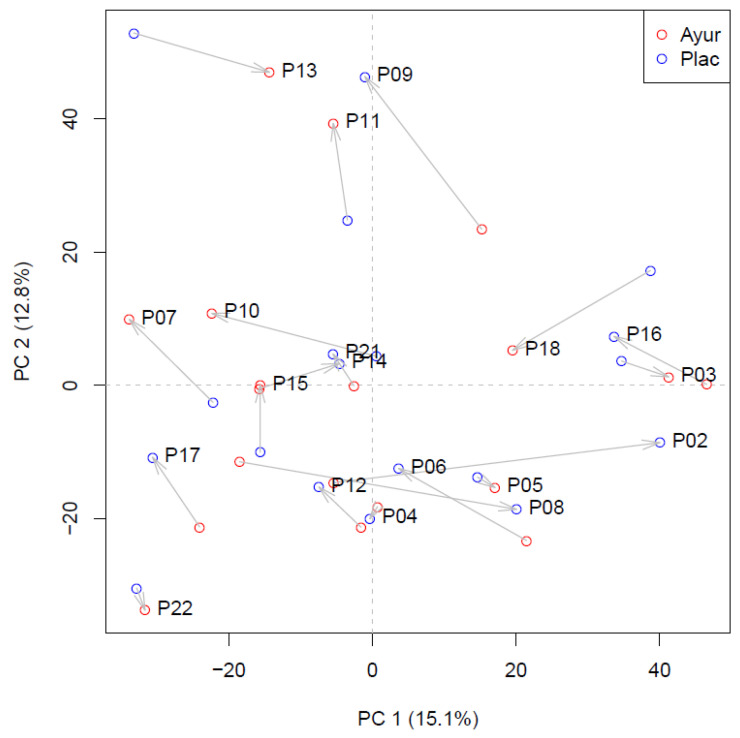
Principal Component Analysis (PCA) score plot of the urine metabolomic data of the subjects. Red symbols indicate treatment, blue symbols indicate placebo. The arrows connect the two symbols for individual subjects, and are pointing from the results of the first stage to the results of the second stage.

Twenty two subjects entered the study. Three subjects dropped out during the study, two due to medical reasons not related to the study and one declined to participate. Baseline characteristics of the remaining 19 subjects are listed in Table 2. Participants did not gain or lose weight during the study (*p* = 0.146) and compliance to the treatment was high; 95.3% of the herbal supplements and 96.0% of the placebo supplements were consumed during the intervention. Principal Component Analysis (PCA) of volunteers based on their 24 h-urine metabolite profiles, representing nearly 3500 compounds, shows that herbal supplementation did not result in a different urine metabolite profile across volunteers when comparing placebo treatment and herbal treatment, but rather indicates large individual differences unrelated to treatment (variable directions of treatment effects in PCA) (Figure 2). Herbal supplementation did not affect plasma levels of liver function enzymes ALAT (*p* = 0.218) and ASAT (*p* = 0.319).

### 3.2. Metabolic Parameters and Differentiated Leukocyte Count

A 4-week herbal intervention did not affect fasting concentrations of HbcA1, plasma glucose, insulin, TG or total cholesterol. The herbal intervention also did not affect the insulin and glucose responses after the OGTT (Table 3). Insulin, HOMA-IR and postprandial insulin levels showed a trend towards an increase after 4-week herbal intervention (*p* = 0.054, *p* = 0.056 and *p* = 0.095 respectively). Leukocyte numbers were not affected by the 4-week herbal intervention (Table 3).

### 3.3. Measures of Vascular Function

One subject was excluded from the vascular measures analysis due to inconsequent intake of blood pressure lowering medication. Three subjects were excluded from the PWA analysis because of bad recordings due to an irregular heartbeat. In total, 18 subjects were analyzed for blood pressure measurements and 15 subjects for PWA measurements. A 4-week herbal intervention did not affect fasting blood pressure outcomes or Aix (Table 3). Both Aix and DBP decreased in response to the OGTT, but the change in OGTT response during the intervention was not different between the herbal and the placebo groups (Table 4).

### 3.4. Urine Metabolites

Applying the Wilcoxon signed-rank test to distinguish between the diets led to 110 metabolites with *p* values below 0.05. Since the number of tested metabolites is large a multiple-testing correction was performed, and the only metabolite having a *p* value below 0.05 after correction was cluster 2943, eluting at 22.97 min with an *m*/*z* value of 275.0519. The elemental formula is C10H16O3N2S2 and it putatively could be a conjugate of isothiocyanate as human metabolite, and a degradation product of glucosinolates present in the herbal mixture. This metabolized plant compound may be used as a biomarker showing the compliance of the volunteers to the herbal supplementation.

### 3.5. Whole Genome Adipose Tissue Gene Expression Analyses

The expression of 499 genes was significantly different after herbal supplementation compared to placebo, of which 221 genes were upregulated and 278 genes downregulated. To elucidate which pathways, signaling routes and networks were regulated by herbal supplementation, we performed a gene set enrichment analysis (GSEA) and clustered gene sets based on overlapping genes within these gene sets. There were 154 gene sets differently regulated after herbal supplementation compared to placebo, of which 147 were upregulated (Supplementary Table S1) and 7 were downregulated (Supplementary Table S2). A large cluster, containing 113 up-regulated gene sets, was involved in inflammation signaling pathways and immune function. The individual changes in expression of the genes that were significantly changed by the herbal supplementation in this cluster are depicted in Supplementary Figure S1. Other up-regulated clusters included leukocyte and integrin interactions (Supplementary Table S1). Two downregulated gene sets were involved in peroxisomal lipid metabolism, but no genes within these gene sets were significantly changed after herbal supplementation compared to placebo (Supplementary Table S2). To identify common upstream regulators of the affected genes, we performed an upstream regulator analysis using Ingenuity Pathway Analysis. Two upstream regulators were identified after herbal supplementation compared to placebo; the BCL2/adenovirus E1B 19 kDa protein-interacting protein 3-like gene (BNIP3L) was activated whereas the Homeobox protein Nkx-2.3 gene (NKX2-3) was inhibited.

## 4. Discussion

The use and popularity of herbal supplements for disease prevention and treatment as an alternative to Western medicine is increasing. This emphasizes the need for a scientific evaluation of the efficacy of these herbal supplements. In the current study we tested the efficacy of the Ayurvedic herbal mixture Mohana Choorna (MC) on a broad range of glucose homeostasis markers, important in the development of T2D in an overweight middle aged population with an impaired glucose tolerance. We showed that a 4-week intervention with herbal supplements showed a trend towards an increased insulin resistance, although this was not significant, as measured by fasting insulin, HOMA-index, and 2 h glucose upon OGTT, but did not affect other markers related to T2D such as dyslipidemia or vascular function. Herbal supplementation up-regulated 113 gene sets in adipose tissue that were involved in inflammation signaling routes and immune function. Out of the nearly 3500 metabolites detected in the 24 h urine samples collected after both intervention periods, none were significantly related to MC intake or glucose homeostasis parameters. A high variation in urine profiles between subjects was observed, maybe reflecting the variation in metabolizing capacity of plant metabolites between individuals.

Human placebo-controlled intervention studies with herbs are rare, but previous in vitro and animal studies indicated that several individual compounds of herbal supplements in our study might have potential beneficial effects on several outcome measures known to be important for the development of T2D. A combination of these herbs, such as the current-used herbal mixture MC, may affect various targets in a mild way, thereby improving metabolic health. However, our results do not support such a hypothesis. On the contrary, the borderline significant increase in fasting insulin, HOMA-index and 2 h glucose after 4-week herbal supplementation, may point towards an insulin desensitizing instead of a beneficial insulin sensitizing effect. However, the trend towards increased insulin may also point towards an increased secretion of insulin by the pancreas. But if herbal supplementation is able to stimulate the pancreas to release insulin, the absence of accompanying lower glucose levels will likely not result in an improvement in metabolic health. Another finding that does not support a beneficial effect of the herbal supplement is the observed increase in gene expression of inflammatory genes in adipose tissue samples. The increase in the expression of inflammation-related gene sets may indicate a prelude towards an inflamed adipose tissue. An increase in inflammation in the adipose tissue has been associated with the development of metabolic diseases, including insulin resistance [48]. Changes in these gene sets and accompanying genes are therefore potential indicators for early disturbances in adipose tissue health after herbal supplementation. As an example, the expression of inflammatory gene sets in adipose tissue are usually increased in obese individuals, whereas caloric restriction is able to lower this expression [49,50,51]. Although the outcomes are relatively consistent, to what extend these MC-induced changes in gene expression profiles are predictive for development of T2D remains to be elucidated.

The observed decrease in DBP and AIX after consumption of the 75 g glucose drink is in accordance with the results of previous studies that have demonstrated a decrease in AIX after consumption of a light breakfast [52], high-fat shakes [36,53] and after both low and high carbohydrate meals [54]. These results may suggest that energy intake leads to neural- and/or hormonal-mediated vascular smooth muscle relaxation.

Limitations of the study.

The design used in this study allowed us to test the efficacy of a 4-week intake of the herbal mixture MC on a variety of health parameters in a placebo-controlled and randomised setting. This study is one of the few trials conducted in humans, more specifically in volunteers with a perturbed glucose tolerance, a potentially important target population for herbal interventions. The compliance with regard to the intake of the capsules was good, and subjects remained weight-stable during the intervention period. One of the points to consider is that longer trials than 4 weeks may be needed to observe any significant effect and that the number of volunteers investigated was relatively small. HbA1c, for example, is a marker for longer term changes in glucose homeostasis that cannot be expected to be changed after a four week treatment. Still, we comprehensively evaluated in a cross-over setting a wide range of glucose homeostasis markers and none of these parameters were beneficially affected by the four week herbal treatment. So far, we have therefore no reason to assume that an increase in sample size may alter current study outcomes in a beneficial direction. In contrast, the borderline significant increase in insulin, in combination with unaffected glucose levels both fasting as well as after the OGTT, may even indicate a perturbing effect on glucose homeostasis. The study population included both males and females but due to the smaller sample size, sub analyzes on sex would be underpowered and could therefore not be performed. We tested the efficacy of a Ayurvedic herbal supplements and did not test Ayurvedic treatment. Treatment is usually personalized and also includes dietary interventions and lifestyle advice. These aspects were not taken into account in the current study set-up. Furthermore, the Ayurvedic herbal supplement investigated in this study is a combination of herbs that might affect the effectiveness of the individual herbs, therefore no conclusions can be drawn about the effects of the individuals herbs. As most studies have been performed in animal and in vitro models with individual herbs, we cannot exclude that we used the most effective amount and combination of the herbs. In addition, most animal models used were hyperglycemic or diabetic, as we used subjects with an impaired glucose tolerance in quite an early phase of development of diabetes, we cannot exclude that the Ayurvedic herbal supplement would have had an effect on diabetic subjects.

## 5. Conclusions

The increased use of alternative Ayurvedic treatments emphasizes the importance of studying the efficacy of herbal supplements in sound placebo-controlled intervention trials. Our findings in overweight middleaged subjects with an impaired glucose tolerance suggest that there is no substantiating evidence to claim that use of the current Ayurvedic herbal supplements as executed in our 4-week trial may beneficially affect glucose homeostasis markers important for the development of T2D in four weeks. In fact, the borderline-significant trend towards an increase in fasting insulin and the up-regulation of inflammatory gene sets in adipose tissue after 4 week herbal supplementation may even point to a deteriorating effect on insulin resistance, glucose handling, HOMA-IR and adipose tissue health. Therefore, the use of such herbal remedies should be handled with care, especially if these remedies are used by subjects already at risk, such as those in our study population.

## Figures and Tables

**Table 1:** Herbs and the amounts (%) used in the herbal supplements and their potentially health effect described in the literature.

Name of Herb (Part Used)	Common Name	% (of Total)	Potential Health Effect (Type of Sudy)
Acorus calamus (rhizome)	Sweet flag	2	Hypoglycaemic, increased glucose metabolism, decrease in TG, FFA, insulin sensitizer (in vitro, animal) [12,13]
Brassica juncea (seed)	Indian mustard	2	Hypoglycaemic, antioxidant (animal) [14,15]
Coriandrum sativum (seed)	Coriander	9	Hypoglycaemic (increased glucose oxidation, increased glyconeogensis), insulin-releasing and insulin-like activity (animal) [4]
Cucurbita ficifolia (fruit)	Siam pumpkin	14	Hypoglycaemic, increased insulin levels, antioxidant (animal) [16,17]
Fagopyrum esculentum (seed)	Buckwheat	7	Insulin sensitizing, antioxidant (animal) [18]
Hordeum vulgare (grain)	Barley	7	Hypoglycaemic (decreased activity of glucose-6-phosphatase), antioxidant (animal/human) [19,20]
Hypericum perforatum (leaf)	St John’s wort	2	Prevention of deleterious effects of diabetes (animal) [21]
Mentha aquatica (whole plant)	Watermint	2	Antioxidant (in vitro) [22]
Mentha crispa (whole plant)	Wrinkled-leaf mint	2	Antioxidant (in vitro) [22]
Mentha piperita (whole plant)	Peppermint	2	Antioxidant (in vitro) [22]
Menyanthes trifoliate (whole plant)	Buckbean	2	
Momordica charantia (fruit)	Bitter melon	14	Inhibition of α-glucosidase, sucrose, maltase, inhibition of porcine pancreatic α-amylase activity and reduced starch hydrolysis (in vitro) [23,24]
Morus alba (leaf and fruit)	White mulberry	2	Hypoglycaemic, hypolipidemic (animal/human) [25,26]
Nasturtium officinale (whole plant)	Watercress	2	Antioxidant (animal) [27]
Nigella sativa (seed)	Fennel flower	5	Hypoglycaemic, increase in serum insulin level (animal) [28]
Origanum vulgare (whole plant)	Oregano	4	Antioxidant (in vitro) [29]
Paspalum scrobiculatum (seed)	Kodo/Kodra millet	7	Hypoglycaemic, increase in serum insulin level, hypolipidemic, increased liver glycogen (animal) [30]
Rosmarinus officinalis (leaf and twig)	Rosemary	2	Hypoglycaemic, increased insulin levels and antioxidant (animal) [31]
Salvia officinalis (leaf and twig)	Common sage	5	Reduction of fasting plasma glucose (decreased gluconeogenesis, increased insulin sensitivity), increased plasma insulin levels (animal) [32,33]
Trigonellafoenum-graecum (seed)	Fenugreek	9	Increased peripheral glucose uptake, insulin-releasing, decreased gluconeogenesis/glycogenolysis, insulin sensitizing (animal/human) [34,35]

**Table 2:** Baseline characteristics of the subjects.

	Mean ± SD (*n* = 19)
Males/females	12/7
Age (y)	64.2 ± 4.5
BMI (kg/m^2^)	30.9 ± 3.9
Fasting glucose (mmol/L)	5.3 ± 0.7
Postprandial (2 h) glucose (mmol/L)	8.9 ± 1.2
Diastolic blood pressure (mmHg)	74 ± 10
Systolic blood pressure (mmHg)	130 ± 24

**Table 3:** Changes in metabolic risk markers after 4-week herbal or placebo supplementation at baseline and in response to an 75 g oral glucose tolerance test in glucose intolerant subjects.

	Intervention					*p* Value	
	Herbal		Placebo		Main		Interaction
	Before (W0)	ΔW4	Before (W0)	ΔW4	Intervention	Time (4 weeks)	Intervention × Time
Markers of glucose metabolism							
*Fasting*							
Glucose (mmol/L)	6.0 ± 0.8	0.0 ± 0.2	5.9 ± 0.8	0.1 ± 0.3	0.410	0.221	0.393
Insulin (mU/L)	13.1 ± 7.3	1.4 ± 4.0	15.6 ± 8.4	−0.4 ± 3.8	0.054	0.269	0.054
HOMA-IR	3.4 ± 1.9	0.4 ± 1.1	4.0 ± 2.2	0.1 ± 1.0	0.051	0.254	0.056
HbA1c (mmol/moL)	40.2 ± 2.6	0.4 ± 0.8	39.9 ± 3.0	0.7 ± 1.1	0.559	<0.001	0.590
Response to 75 g OGTT							
2 h glucose (mmol/L)	7.9 ± 2.3	0.4 ± 1.6	8.4 ± 2.3	−0.2 ± 1.0	0.052	0.370	0.057
2 h insulin (mU/L)	76.3 ± 40.9	10.9 ± 24.6	90.2 ± 52.1	−3.7 ± 26.7	0.084	0.153	0.095
iAUC Δ glucose (AU)	277 ± 197	20 ± 77	333 ± 178	−13 ± 81	0.162	0.389	0.167
iAUC Δ insulin (AU)	5873 ± 3602	569 ± 2141	6692 ± 4666	84 ± 2018	0.595	0.236	0.484
Markers of vascular health							
SBP (mmHg)	126 ± 17	0 ± 8	127 ± 18	0 ± 9	0.811	0.996	0.807
DBP (mmHg)	73 ± 10	0 ± 4	73 ± 8	0 ± 4	0.970	0.576	0.980
AIX (%)	25 ± 6	0 ± 3	25 ± 6	1 ± 4	0.354	0.595	0.444
Total cholesterol (mmol/L)	5.8 ± 0.9	−0.1 ± 0.4	5.8 ± 1.1	0.1 ± 0.5	0.255	0.678	0.255
TG (mmol/L)	1.6 ± 0.5	0.0 ± 0.4	1.7 ± 0.7	0.0 ± 0.4	0.618	0.804	0.618
Leucocytes 10^9^ (#/l)	5.6 ± 13	0.2 ± 0.9	6.4 ± 2.2	−0.4 ± 1.2	0.099	0.154	0.141

Values are mean ± SD, delta values are compared to baseline, *n* = 19, for SBP and DBP *n=* 18 and for AIX *n* = 15. *P* values were calculated by using linear mixed models for repeated measures. I, intervention effect; T, time effect; I x T, intervention x time interaction; OGTT, oral glucose tolerance test; iAUC, incremental area under the curve; SBP, systolic blood pressure; DBP, diastolic blood pressure; AIX, augmentation index; TG, triglycerides.

**Table 4:** Changes in glucose, insulin and vascular function in response to a 75 g oral glucose tolerance test after 4-week herbal or placebo supplementation in glucose-intolerant subjects.

		Before	(W0)			After	(W4)			*p* Value
										Change in overall response
	I	ΔT30	ΔT60	ΔT90	ΔT120	ΔT30	ΔT60	ΔT90	ΔT120	between interventions
Glucose	Herbal	2.6 ± 1.3	3.6 ± 2.1	2.6 ± 2.9	1.9 ± 2.2	2.9 ± 1.3	3.8 ± 2.7	3.0 ± 2.6	2.7 ± 2.3	0.49
(mmol/L)	Placebo	3.0 ± 1.5	3.7 ± 2.2	3.1 ± 2.3	2.5 ± 1.9	3.1 ± 1.4	3.3 ± 2.3	2.9 ± 2.9	2.0 ± 2.3	
Insulin	Herbal	34 ± 32	72 ± 64	61 ± 34	63 ± 37	39 ± 36	76 ± 62	73 ± 56	82 ± 48	0.44
(mU/L)	Placebo	43 ± 46	71 ± 54	77 ± 52	75 ± 49	51 ± 42	68 ± 40	71 ± 44	70 ± 41	
SBP	Herbal	-	1 ± 10	-	−3 ± 12	-	−2 ± 5	-	−3 ± 8	0.40
(mmHg)	Placebo	-	0 ± 9	-	−5 ± 9	-	0 ± 13	-	1 ± 16	
DBP	Herbal	-	−2 ± 6	-	−3 ± 5	-	−3 ± 4	-	−4 ± 3	0.78
(mmHg)	Placebo	-	−2 ± 5	-	−4 ± 4	-	−3 ± 4	-	−3 ± 5	
AIX	Herbal	-	−5 ± 4	-	−5 ± 4	-	−5 ± 3	-	−5 ± 4	0.79
(%)	Placebo	-	−5 ± 4	-	−5 ± 4	-	−5 ± 5	-	−5 ± 4	

Values are mean ± SD, delta values are compared to baseline (T0), *n* = 19 for glucose and Insulin, *n=* 18 for SBP and DBP and *n* = 15 for AIX. *P* values of the change in response between interventions were calculated by using linear mixed models for repeated measures on delta values (week4–week0) for each postprandial time point by using the interaction “intervention*postprandial time point” as a fixed effect. SBP, systolic blood pressure; DBP, diastolic blood pressure; AIX, augmentation index.

## Data Availability

Microarray data are registered as GSEXXXX in the Gene Expression Omnibus.

## Supplementary Materials

The following are available online at https://www.mdpi.com/2072-6643/13/1/260/s1, Figure S1: Expression heat map of significantly changed genes after 4 week herbal supplementation to placebo from the up-regulated gene set cluster “inflammation”. Table S1: Upregulated gene sets after 4-week herbal supplementation compared to placebo in adipose tissue samples from overweight subjects with an impaired glucose tolerance. Table S2: Upregulated gene sets after 4-week herbal supplementation compared to placebo in adipose tissue samples from overweight subjects with an impaired glucose tolerance.
