# Supplementary material for: Ayurvedic Herbal Preparation Supplementation Does Not Improve Metabolic Health in Impaired Glucose Tolerance Subjects; Observations from a Randomised Placebo Controlled Trial
Source: Nutrients. 2021 Jan 18;13(1):260. doi: 10.3390/nu13010260 (PMC7830190; doi:10.3390/nu13010260)
Supplement: Supplementary file 1 [file nutrients-13-00260-s001.pdf]

Supplementary Materials

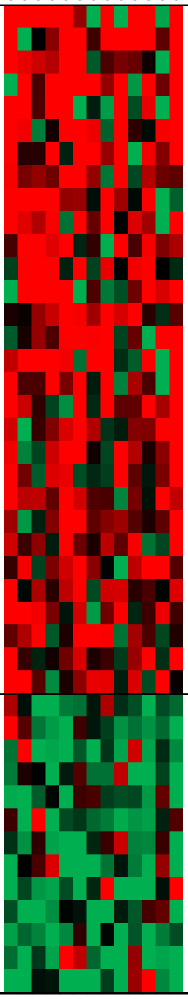

gene name	Entrez ID	description	SlSlSlSlSlSlSlSlSlSlSlSlSl	verage SLI	p-value
TNFSF13	8741	tumor necrosis factor (ligand) superfamily, member 13		0.69	0.03
OR10S1	219873	olfactory receptor, family 10, subfamily S, member 1		0.30	0.00
NCF4	4689	neutrophil cytosolic factor 4, 40kDa		0.26	0.01
DEFB124	245937	defensin, beta 124		0.26	0.01
PTAFR	5724	platelet-activating factor receptor		0.25	0.04
IFITM2	10581	interferon induced transmembrane protein 2		0.23	0.01
KLK2	3817	kallikrein-related peptidase 2		0.22	0.02
CD14	929	CD14 molecule		0.22	0.01
CCL22	6367	chemokine (C-C motif) ligand 22		0.21	0.04
PTPRCAP	5790	protein tyrosine phosphatase, receptor type, C-associated protein		0.21	0.02
HLA-DPB1	3115	major histocompatibility complex, class II, DP beta 1		0.21	0.03
P2RY6	5031	pyrimidinergic receptor P2Y, G-protein coupled, 6		0.20	0.01
NFATC2	4773	nuclear factor of activated T-cells, cytoplasmic, calcineurin-dependent 2		0.20	0.05
GPR25	2848	G protein-coupled receptor 25		0.20	0.01
TUBB2A	7280	tubulin, beta 2A class IIa		0.19	0.03
TRAF1	7185	TNF receptor-associated factor 1		0.19	0.02
TGFB1	7040	transforming growth factor, beta 1		0.19	0.04
BDKRB2	624	bradykinin receptor B2		0.17	0.02
CD79B	974	CD79b molecule, immunoglobulin-associated beta		0.16	0.03
TAP2	6891	transporter 2, ATP-binding cassette, sub-family B (MDR/TAP)		0.16	0.02
PLCG2	5336	phospholipase C, gamma 2 (phosphatidylinositol-specific)		0.15	0.04
TMEM173	340061	transmembrane protein 173		0.15	0.01
IRF9	10379	interferon regulatory factor 9		0.15	0.02
PSMB8	5696	proteasome (prosome, macropain) subunit, beta type, 8		0.14	0.03
SH2B1	25970	SH2B adaptor protein 1		0.13	0.04
CD74	972	CD74 molecule, major histocompatibility complex, class II invariant chain		0.13	0.01
GNG13	51764	guanine nucleotide binding protein (G protein), gamma 13		0.13	0.02
MYH7B	57644	myosin, heavy chain 7B, cardiac muscle, beta		0.12	0.05
TNRC6B	23112	trinucleotide repeat containing 6B		0.11	0.04
DDX41	51428	DEAD (Asp-Glu-Ala-Asp) box polypeptide 41		0.11	0.05
ECSIT	51295	ECSIT signalling integrator		-0.12	0.04
BRPF3	27154	bromodomain and PHD finger containing, 3		-0.13	0.04
PIK3R2	5296	phosphoinositide-3-kinase, regulatory subunit 2 (beta)		-0.13	0.03
ORAI1	84876	ORAI calcium release-activated calcium modulator 1		-0.13	0.03
FGF13	2258	fibroblast growth factor 13		-0.13	0.02
PDE11A	50940	phosphodiesterase 11A		-0.13	0.04
GNAI1	2770	guanine nucleotide binding protein (G protein), alpha inhibiting activity polypeptide 1		-0.14	0.04
FGF12	2257	fibroblast growth factor 12		-0.17	0.02
SHC3	53358	SHC (Src homology 2 domain containing) transforming protein 3		-0.18	0.04
TNFRSF10D	8793	tumor necrosis factor receptor superfamily, member 10d, decoy with truncated death domain		-0.18	0.01
CCNE1	898	cyclin E1		-0.21	0.00
FIGF	2277	c-fos induced growth factor (vascular endothelial growth factor D)		-0.21	0.03
FGF10	2255	fibroblast growth factor 10		-0.22	0.01

Supplementary Figure S1: Expression heat map of significantly changed genes after 4 week herbal supplementation to placebo from the up-regulated gene set cluster 'inflammation'. Expression changes are indicated as individual signal-log-ratios (SLR) of herbal versus placebo from adipose tissue samples of 13 overweight subjects with an impaired glucose tolerance. Down-regulation or up-regulation of gene expression is presented on a colour scale ranging from green (down-regulated, SLR ≤ -0.25) to red (up-regulated, SLR ≥ 0.25).

Supplementary Table S1: Upregulated gene sets after 4-week herbal supplementation compared to placebo in adipose tissue samples from overweight subjects with an impaired glucose tolerance.

NAME	SIZE	NES	FDR q-val
**Immune function**			
FCERI.MEDIATED.CA.2.MOBILIZATION	29	2.5	0.00
KEGG_NATURAL.KILLER.CELL.MEDIATED.CYTOTOXICITY	91	2.3	0.00
GENERATION.OF.SECOND.MESSENGER.MOLECULES	29	2.2	0.01
THE.ROLE.OF.NEF.IN.HIV.1.REPLICATION.AND.DISEASE.PATHOGENESIS	25	2.2	0.01
ANTIGEN.ACTIVATES.B.CELL.RECEPTOR.BCR.LEADING.TO.GENERATION.OF.SECOND.MESSENGERS	33	2.1	0.01
KEGG_ANTIGEN.PROCESSING.AND.PRESENTATION	54	2.1	0.01
KEGG_ALLOGRAFT.REJECTION	21	2.1	0.01
WP2732.INTERLEUKIN.2.SIGNALING	23	2.1	0.01
WP2694.DAP12.INTERACTIONS	32	2.1	0.01
KEGG_LEISHMANIASIS	55	2.1	0.01
KEGG_AUTOIMMUNE.THYROID.DISEASE	22	2.1	0.01

WP1826.GPVI.MEDIATED.ACTIVATION.CASCADE	19	2.1	0.01
INTERFERON.ALPHA.BETA.SIGNALING	49	2.0	0.01
BIOC_BCRPATHWAY	33	2.0	0.01
WP23.B.CELL.RECEPTOR.SIGNALING.PATHWAY	87	2.0	0.01
KEGG_GRAFT.VERSUS.HOST.DISEASE	24	2.0	0.01
KEGG_B.CELL.RECEPTOR.SIGNALING.PATHWAY	68	2.0	0.01
WP69.TCR.SIGNALING.PATHWAY	81	2.0	0.01
KEGG_TYPE.I.DIABETES.MELLITUS	25	2.0	0.01
BIOC_FMLPPATHWAY	32	2.0	0.01
WP1840.INTERLEUKIN.3.5.AND.GM.CSF.SIGNALING	31	2.0	0.01
WP1835.INTERFERON.ALPHA.BETA.SIGNALING	29	2.0	0.01
KEGG_NF.KAPPA.B.SIGNALING.PATHWAY	82	2.0	0.02
BIOC_CASPASEPATHWAY	21	1.9	0.02
WP1799.COSTIMULATION.BY.THE.CD28.FAMILY	39	1.9	0.02
INFLAMMASOMES	16	1.9	0.02
KEGG_LEUKOCYTE.TRANSENDOTHELIAL.MIGRATION	90	1.9	0.02
PHOSPHORYLATION.OF.CD3.AND.TCR.ZETA.CHAINS	17	1.9	0.02
FCERI.MEDIATED.MAPK.ACTIVATION	43	1.9	0.02
KEGG_STAPHYLOCOCCUS.AUREUS.INFECTION	39	1.9	0.02
EFFECTS.OF.PIP2.HYDROLYSIS	21	1.9	0.03
KEGG_TUBERCULOSIS	135	1.9	0.03
WP2796.CLASS.I.MHC.MEDIATED.ANTIGEN.PROCESSING.AMP.PRESENTATION	77	1.9	0.03
WP2775.TOLL.LIKE.RECEPTORS.CASCADES	22	1.9	0.03
BIOC_NKCELLSPATHWAY	17	1.9	0.03
CYTOKINE.SIGNALING.IN.IMMUNE.SYSTEM	237	1.9	0.03
INTERFERON.SIGNALING	140	1.9	0.03
KEGG_T.CELL.RECEPTOR.SIGNALING.PATHWAY	87	1.9	0.02
WP2746.SIGNALING.BY.THE.B.CELL.RECEPTOR.BCR.	100	1.9	0.03
BIOC_TCRPATHWAY	41	1.9	0.03
TRANSLOCATION.OF.ZAP.70.TO.IMMUNOLOGICAL.SYNAPSE	15	1.9	0.03
KEGG_INTESTINAL.IMMUNE.NETWORK.FOR.IGA.PRODUCTION	30	1.8	0.03
KEGG_VIRAL.MYOCARDITIS	47	1.8	0.03
GPVI.MEDIATED.ACTIVATION.CASCADE	43	1.8	0.03
NEF.MEDIATES.DOWN.MODULATION.OF.CELL.SURFACE.RECEPTORS.BY.RECRUITING. THEM.TO.CLATHRIN.ADAPTERS	18	1.8	0.04
WP1836.INTERFERON.GAMMA.SIGNALING	25	1.8	0.04
BIOC_ECMPATHWAY	21	1.8	0.04
WP286.IL.3.SIGNALING.PATHWAY	45	1.8	0.05
INTERFERON.GAMMA.SIGNALING	57	1.8	0.05
WP2583.T.CELL.RECEPTOR.AND.CO.STIMULATORY.SIGNALING	27	1.8	0.05
INNATE.IMMUNE.SYSTEM	463	1.8	0.05
KEGG_PHAGOSOME	124	1.7	0.05
BIOC_FCER1PATHWAY	36	1.7	0.05
BIOC_EPOPATHWAY	18	1.7	0.06
NRAGE.SIGNALS.DEATH.THROUGH.JNK	39	1.7	0.06
BIOC_IL2PATHWAY	21	1.7	0.06
WP455.GPCRS.CLASS.A.RHODOPSIN.LIKE	111	1.7	0.06
WP2719.FCGAMMA.RECEPTOR.FCGR.DEPENDENT.PHAGOCYTOSIS.	50	1.7	0.06
TCR.SIGNALING	54	1.7	0.06
INTERLEUKIN.3.5.AND.GM.CSF.SIGNALING	40	1.7	0.06
ANTIGEN.PROCESSING.CROSS.PRESENTATION	69	1.7	0.06
WP2759.FC.EPSILON.RECEPTOR.FCERI.SIGNALING.	62	1.7	0.07
REGULATION.OF.ACTIN.DYNAMICS.FOR.PHAGOCYTIC.CUP.FORMATION	57	1.7	0.06
KEGG_INFLAMMATORY.BOWEL.DISEASE.IBD.	42	1.7	0.06
PD.1.SIGNALING	19	1.7	0.07
WP2328.ALLOGRAFT.REJECTION	53	1.7	0.07
KEGG_OSTEOCLAST.DIFFERENTIATION	113	1.7	0.08

NUCLEOTIDE.BINDING.DOMAIN.LEUCINE.RICH.REPEAT.CONTAINING.RECEPTOR.NLR.SIGNALING.PATHWAYS	43	1.7	0.09
BIOC_RHOPATHWAY	28	1.7	0.09
WP1927.TCR.SIGNALING	49	1.7	0.09
PLATELET.ACTIVATION.SIGNALING.AND.AGGREGATION	183	1.6	0.09
WP384.APOPTOSIS.MODULATION.BY.HSP70	19	1.6	0.10
KEGG_INFLUENZA.A	138	1.6	0.11
FCGAMMA.RECEPTOR.FCGR.DEPENDENT.PHAGOCYTOSIS	80	1.6	0.11
WP2684.HOST.INTERACTIONS.OF.HIV.FACTORS	114	1.6	0.11
WP2507.NANOMATERIAL.INDUCED.APOPTOSIS	20	1.6	0.11
ER.PHAGOSOME.PATHWAY	57	1.6	0.11
KEGG_FC.GAMMA.R.MEDIATED.PHAGOCYTOSIS	84	1.6	0.12
KEGG_PHOSPHATIDYLINOSITOL.SIGNALING.SYSTEM	71	1.6	0.12
SIGNALING.BY.INTERLEUKINS	99	1.6	0.12
HOST.INTERACTIONS.OF.HIV.FACTORS	119	1.6	0.12
KEGG_MEASLES	108	1.6	0.12
KEGG_ASTHMA	15	1.6	0.12
KEGG_FC.EPSILON.RI.SIGNALING.PATHWAY	59	1.6	0.14
BIOC_ERK5PATHWAY	16	1.6	0.14
BIOC_IL7PATHWAY	15	1.6	0.15
WP2752.MYD88.INDEPENDENT.CASCADE.	22	1.6	0.15
BIOC_NGFPATHWAY	17	1.5	0.16
ROLE.OF.PHOSPHOLIPIDS.IN.PHAGOCYTOSIS	25	1.5	0.16
BIOC_INTEGRINPATHWAY	32	1.5	0.17
KEGG_TOXOPLASMOSIS	99	1.5	0.17
WP314.FAS.PATHWAY.AND.STRESS.INDUCTION.OF.HSP.REGULATION	35	1.5	0.17
KEGG_CHEMOKINE.SIGNALING.PATHWAY	148	1.5	0.17
WP231.TNF.ALPHA.SIGNALING.PATHWAY	82	1.5	0.17
TRAF6.MEDIATED.IRF7.ACTIVATION	18	1.5	0.18
ACTIVATION.OF.IRF3.IRF7.MEDIATED.BY.TBK1.IKK.EPSILON	16	1.5	0.18
WP395.IL.4.SIGNALING.PATHWAY	49	1.5	0.18
INTERLEUKIN.2.SIGNALING	41	1.5	0.19
SEMA4D.INDUCED.CELL.MIGRATION.AND.GROWTH.CONE.COLLAPSE	23	1.5	0.19
AUF1.HNRNP.D0.DESTABILIZES.MRNA	50	1.5	0.19
WP2808.TNF.ALPHA.SIGNALING.PATHWAY	82	1.5	0.19
WP185.INTEGRIN.MEDIATED.CELL.ADHESION	88	1.5	0.19
ANTIGEN.PRESENTATION.FOLDING.ASSEMBLY.AND.PEPTIDE.LOADING.OF.CLASS.I.MHC	21	1.5	0.20
SIGNALING.BY.CONSTITUTIVELY.ACTIVE.EGFR	17	1.5	0.21
WP1433.NOD.PATHWAY	28	1.5	0.21
GPCR.DOWNSTREAM.SIGNALING	339	1.5	0.21
CELL.DEATH.SIGNALLING.VIA.NRAGE.NRIF.AND.NADE	54	1.5	0.21
KEGG_REGULATION.OF.ACTIN.CYTOSKELETON	172	1.5	0.22
PLATELET.DEGRANULATION	65	1.5	0.24
BIOC_GHPATHWAY	23	1.5	0.24
CHEMOKINE.RECEPTORS.BIND.CHEMOKINES	32	1.4	0.25
SYNTHESIS.OF.IP3.AND.IP4.IN.THE.CYTOSOL	21	1.4	0.25
TOLL.LIKE.RECEPTORS.CASCADES	133	1.4	0.25
**Translation**			
WP1813.EUKARYOTIC.TRANSLATION.TERMINATION	94	1.9	0.02
WP1811.EUKARYOTIC.TRANSLATION.ELONGATION	98	1.9	0.02
WP477.CYTOPLASMIC.RIBOSOMAL.PROTEINS	98	1.8	0.03
WP1812.EUKARYOTIC.TRANSLATION.INITIATION	122	1.7	0.06
WP2737.SRP.DEPENDENT.COTRANSLATIONAL.PROTEIN.TARGETING.TO.MEMBRANE	113	1.7	0.07
WP2710.NONSENSE.MEDIATED.DECAY	116	1.7	0.06
WP2683.INFLUENZA.LIFE.CYCLE	193	1.6	0.12
PEPTIDE.CHAIN.ELONGATION	67	1.5	0.21
**Notch signaling**			

PRE.NOTCH.TRANSCRIPTION.AND.TRANSLATION	30	1.8	0.03
PRE.NOTCH.EXPRESSION.AND.PROCESSING	45	1.8	0.05
WP2786.PRE.NOTCH.EXPRESSION.AND.PROCESSING	30	1.6	0.15
**Infection**			
WP2272.PATHOGENIC.ESCHERICHIA.COLI.INFECTION	50	2.0	0.01
KEGG_PATHOGENIC.ESCHERICHIA.COLI.INFECTION	49	2.0	0.01
**Immunoregulation**			
IMMUNOREGULATORY.INTERACTIONS.BETWEEN.A.LYMPHOID.AND.A.NON.LYMPHOID.CELL	54	2.5	0.00
WP1829.IMMUNOREGULATORY.INTERACTIONS.BETWEEN.A.LYMPHOID.AND.A.NON.LYMPHOID.CELL	43	2.2	0.00
**Cytokine receptor**			
BIOC_NKTPATHWAY	19	2.0	0.01
KEGG_CYTOKINE.CYTOKINE.RECEPTOR.INTERACTION	155	1.5	0.19
**Interactions with vascular wall**			
WP1794.CELL.SURFACE.INTERACTIONS.AT.THE.VASCULAR.WALL	71	1.9	0.03
CELL.SURFACE.INTERACTIONS.AT.THE.VASCULAR.WALL	81	1.8	0.05
**Integrin surface interactions**			
WP1833.INTEGRIN.CELL.SURFACE.INTERACTIONS	46	1.6	0.11
INTEGRIN.CELL.SURFACE.INTERACTIONS	52	1.5	0.21
**Other**			
WP2023.CELL.DIFFERENTIATION.META	20	2.1	0.01
KEGG_PRIMARY.IMMUNODEFICIENCY	29	2.0	0.01
KEGG_HEMATOPOIETIC.CELL.LINEAGE	62	2.0	0.01
WP453.INFLAMMATORY.RESPONSE.PATHWAY	23	1.8	0.05
KEGG_LEGIONELLOSIS	50	1.7	0.06
WP2849.HEMATOPOIETIC.STEM.CELL.DIFFERENTIATION	32	1.7	0.07
WP2806.HUMAN.COMPLEMENT.SYSTEM	70	1.7	0.09
WP2784.BINDING.AND.UPTAKE.OF.LIGANDS.BY.SCAVENGER.RECEPTORS	33	1.6	0.13
WP585.INTERFERON.TYPE.I.SIGNALING.PATHWAYS	56	1.6	0.13
WP2742.SIGNALING.BY.TGF.BETA.RECEPTOR.COMPLEX	34	1.5	0.16
KEGG_GALACTOSE.METABOLISM	22	1.5	0.16
KEGG_AMYOTROPHIC.LATERAL.SCLEROSIS.ALS.	37	1.5	0.22
KEGG_MICRORNAS.IN.CANCER	159	1.5	0.23

Ranking based on normalised enrichment score (NES). FDRQ<0.25 was considered significant. Gene sets were clustered by Cytoscape based on an overlap coefficient cut-off of 0.5. Abbreviations: KEGG, Kyoto Encyclopedia of Genes and Genomes database; NCI, Nature Pathway Interaction database; REACT, Reactome knowledgebase; WIP_HS, WikiPathways Homo Sapiens.

Supplementary Table S2: Downregulated gene sets after 4-week herbal supplementation compared to placebo in adipose tissue samples from overweight subjects with an impaired glucose tolerance.

NAME	SIZE	NES	FDR q-val
**Peroxisome**			
WP1878.PEROXISOMAL.LIPID.METABOLISM	19	-1.9	0.15
KEGG_PEROXISOME	70	-1.8	0.24
**Cell growth**			
G0.AND.EARLY.G1	20	-2.1	0.03
WP2446.RB.IN.CANCER	72	-1.8	0.21
**Other**			
KEGG_VALINE.LEUCINE.AND.ISOLEUCINE.DEGRADATION	42	-2.0	0.02
NUCLEAR.RECEPTOR.TRANSCRIPTION.PATHWAY	37	-1.9	0.14
KINESINS	16	-1.7	0.22

Ranking based on normalised enrichment score (NES). FDRQ<0.25 was considered significant. Genesets were clustered by Cytoscape based on an overlap coefficient cut-off of 0.5. Abbreviations: KEGG, Kyoto Encyclopedia of Genes and Genomes database; NCI, Nature Pathway Interaction database; REACT, Reactome knowledgebase; WIP_HS, WikiPathways Homo Sapiens.
